# Supplementary material for: Assessing the risk of early-onset dementia within 5 years of cancer diagnosis Running Title: Early-onset dementia after cancer diagnosis
Source: medRxiv. 2026 Feb 15:2026.02.12.26346204. Preprint. [Version 1] doi: 10.64898/2026.02.12.26346204 (PMC12919094; doi:10.64898/2026.02.12.26346204)
Supplement: Supplement 1 [file media-1.pdf]

Supplementary Methods

We matched beneficiaries by cancer status for incident diagnoses of lung, breast, colon, and prostate cancers. We used incidence density matching with replacement to match beneficiaries with cancer to beneficiaries without cancer at the date of cancer diagnosis. Beneficiaries with cancer diagnosis after b_0 (the exposed) were sorted ascendingly by date of diagnosis (Figure S1). Using the sorted data, the matching pool for exposed beneficiary *i* was composed of all the unexposed at the time of cancer diagnosis t_i (index date), regardless of future exposure. Any unexposed in the matching pool who had a dementia diagnosis, died, turned 65 years old, or ended Medicaid enrollment before t_i was removed. The beneficiaries in the matching pool were matched to the exposed by age at b_0 +/- 2 years, sex (lung and colon cancers), race/ethnicity, state, and year at b_0 +/- 2 years. Each exposed and all its matches formed a cluster. For each cluster *i*, the index date, t_i, was set as the new analytic baseline. Matches with any cancer or dementia diagnosis prior to t_i were excluded.

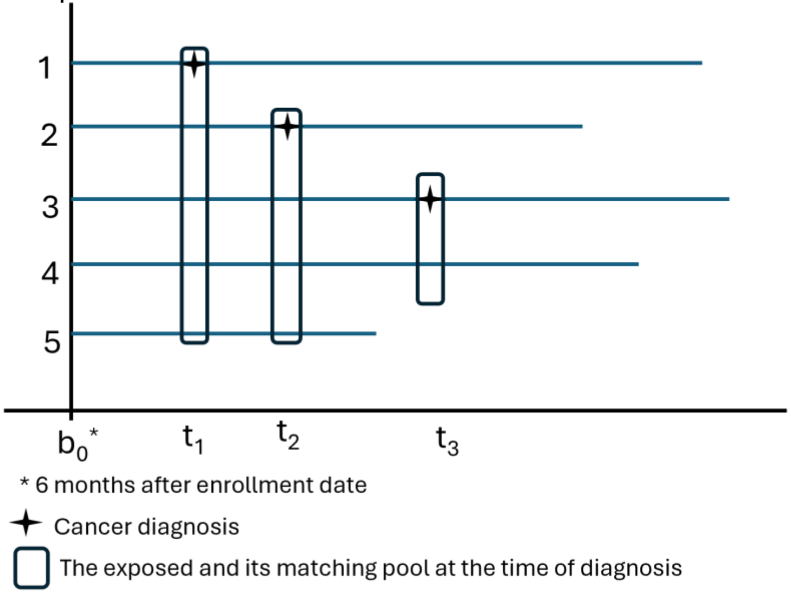

Figure S1. Diagram illustrating the steps on how the matching pool for the exposed group were obtained.

Supplementary tables and figures

Table S1. List of ICD codes used in the study

Variable	ICD-9 Code[a]	ICD-10 Code[a]
**Cancers**		
Lung	162.2-162.5, 162.8, 162.9	C34.x
Colon	153.x	C18.x
Breast	174.x, 175.x	C50.x
Prostate	185.x	C61.x
Any cancer	140.x-195.x, 199.x-208.x	C00.x-C43.x, C45.x-C76.x, C80.x-C96.x
**Dementia**	046.19, 290.4x, 294.0, 294.1x, 294.2x, 294.8, 294.9, 331.0, 331.1x, 331.5, 331.6, 331.7, 331.8x, 331.82, 438.0, 781.8	A81.00, A81.09, F01.x, F02.8x, F03.9x, F04, G30.x, G31.0x, G31.83, G31.85, G91.2, G94, I69.x, R41.4
**Charlson comorbidities**		
Myocardial infarction	410.x, 412.x	I21.x, I22.x, I25.2
Congestive heart failure	398.91, 402.01, 402.11, 402.91, 404.01, 404.03, 404.11, 404.13, 404.91, 404.93, 425.4-425.9, 428.x	I09.9, I11.0, I13.0, I13.2, I25.5, I42.0, I42.5-I42.9, I43.x, I50.x, P29.0
Peripheral vascular disease	093.0, 437.3, 440.x, 441.x, 443.1-443.9, 447.1, 557.1, 557.9, V43.4	I70.x, I71.x, I73.1, I73.8, I73.9, I77.1, I79.0, I79.2, K55.1, K55.8, K55.9, Z95.8, Z95.9
Cerebrovascular disease	362.34, 430.x-438.x	G45.x, G46.x, H34.0, I60.x-I69.x
Chronic pulmonary disease	416.8, 416.9, 490.x-505.x, 506.4, 508.1, 508.8	I27.8, I27.9, J40.x-J47.x, J60.x-J67.x, J68.4, J70.1, J70.3
Rheumatic disease	446.5, 710.0-710.4, 714.0-714.2, 714.8, 725.x	M05.x, M06.x, M31.5, M32.x-M34.x, M35.1, M35.3, M36.0
Peptic ulcer disease	531.x-534.x	K25.x-K28.x
Mild liver disease	070.22, 070.23, 070.32, 070.33, 070.44, 070.54, 070.6, 070.9, 570.x, 571.x, 573.3, 573.4, 573.8, 573.9, V42.7	B18.x, K70.0-K70.3, K70.9, K71.3-K71.5, K71.7, K73.x, K74.x, K76.0, K76.2-K76.4, K76.8, K76.9, Z94.4
Diabetes without chronic complication	250.0-250.3, 250.8, 250.9	E10.0, E10.I, E10.6, E10.8, E10.9, E11.0, E11.1, E11.6, E11.8, E11.9, E12.0, E12.1, E12.6, E12.8, E12.9, E13.0, E13.1, E13.6, E13.8, E13.9, E14.0, E14.1, E14.6, E14.8, E14.9

Diabetes with chronic complication	250.4-250.7	E10.2-E10.5, E10.7, E11.2-E11.5, E11.7, E12.2-E12.5, E12.7, E13.2-E13.5, E13.7, E14.2-E14.5, E14.7
Hemiplegia or paraplegia	334.1, 342.x, 343.x, 344.0-344.6, 344.9	G04.1, G11.4, G80.1, G80.2, G81.x, G82.x, G83.0-G83.4, G83.9
Renal disease	403.01, 403.11, 403.91, 404.02, 404.03, 404.12, 404.13, 404.92, 404.93, 582.x, 583.0-583.7, 585.x, 586.x, 588.0, V42.0, V45.1, V56.x	I12.0, I13.1, N03.2-N03.7, N05.2-N05.7, N18.x, N19.x, N25.0, Z49.0-Z49.2, Z94.0, Z99.2
Moderate or severe liver disease	456.0-456.2, 572.2-572.8	I85.0, I85.9, I86.4, I98.2, K70.4, K71.1, K72.1, K72.9, K76.5, K76.6, K76.7
HIV	042, 079.53, 795.71, V08	B20, B97.35, R75, Z21

[a] A code ending with ".x" indicates a wildcard. Any number could appear after the decimal place.

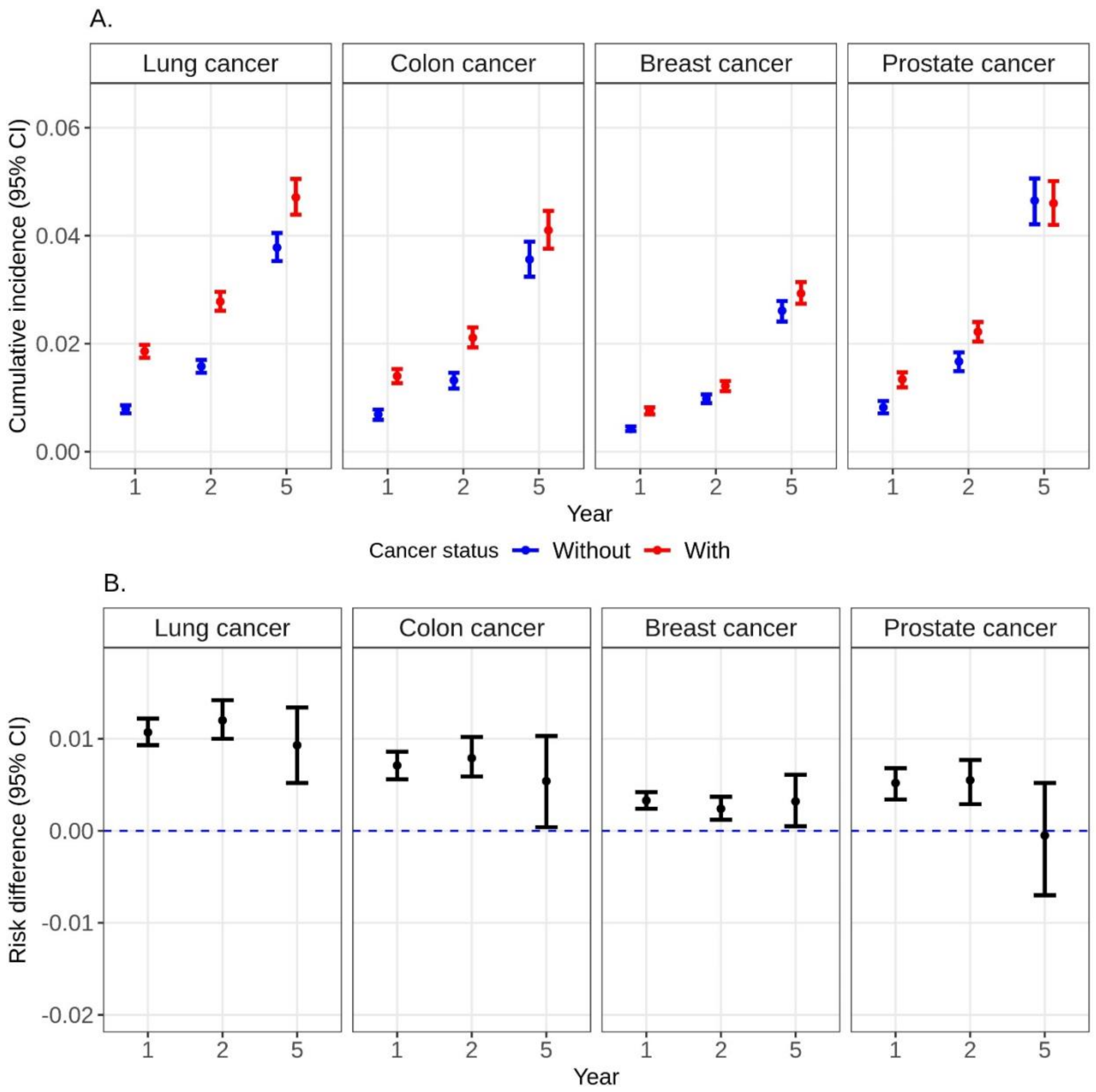

Figure S2. A. Crude cumulative incidence (risk%) and B. crude risk difference of dementia at years 1, 2, and 5 after baseline among matched Medicaid beneficiaries by cancer status.

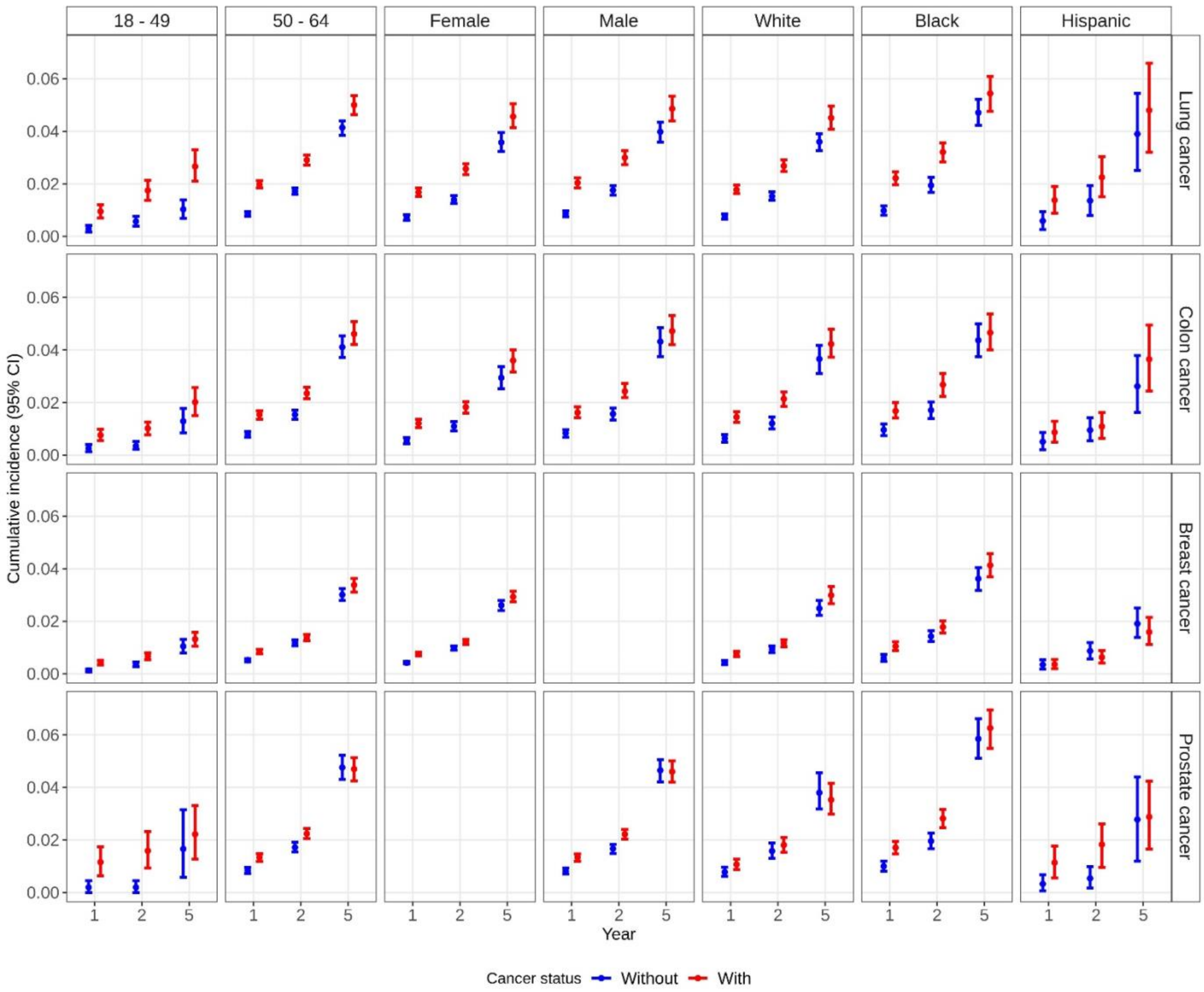

Figure S3. Crude cumulative incidence (risk%) of dementia at years 1, 2, and 5 after baseline among matched Medicaid beneficiaries by cancer status, age, sex, and race/ethnicity.

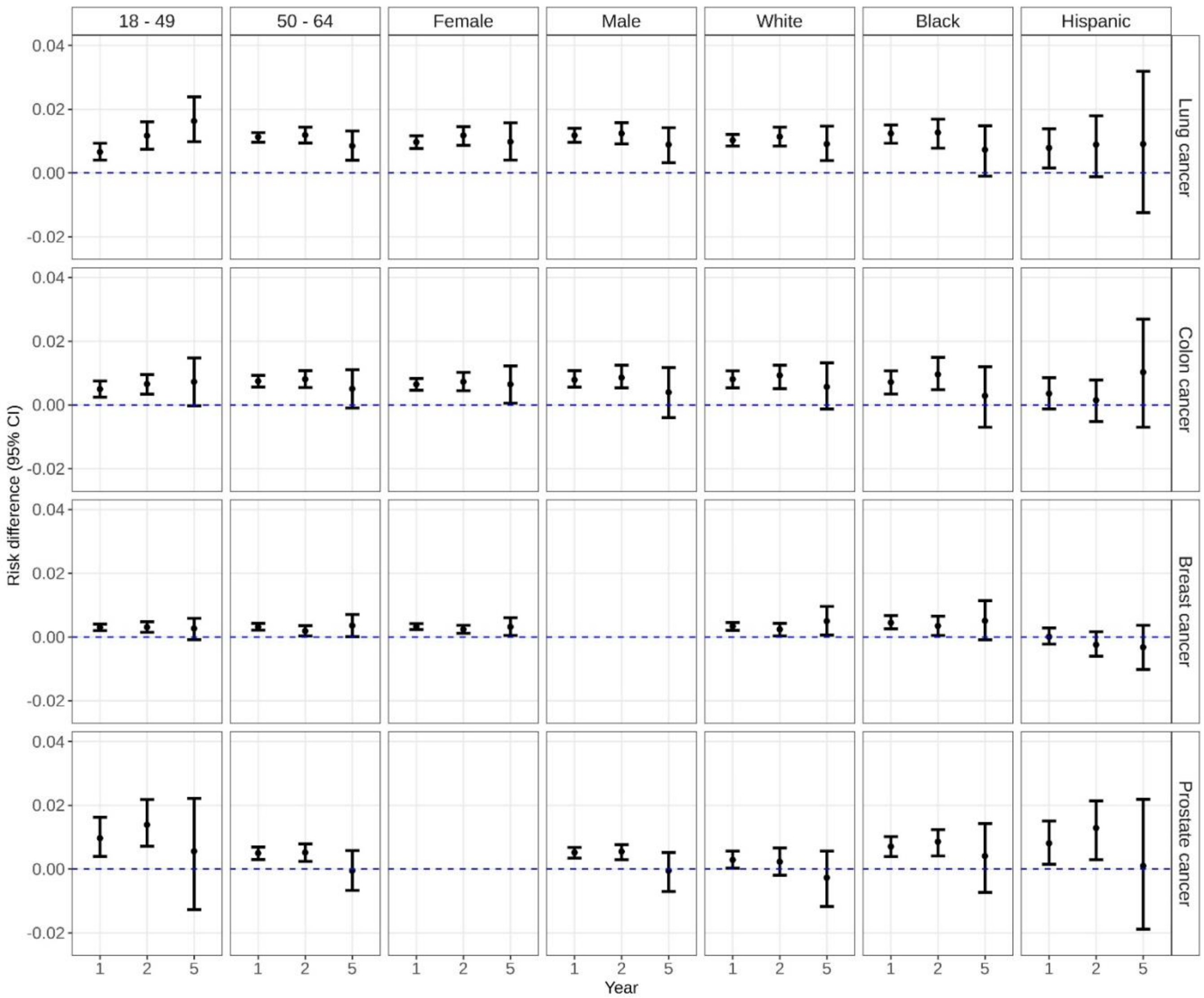

Figure S4. Crude risk difference of dementia at years 1, 2, and 5 after baseline among matched Medicaid beneficiaries by cancer status, age, sex, and race/ethnicity.

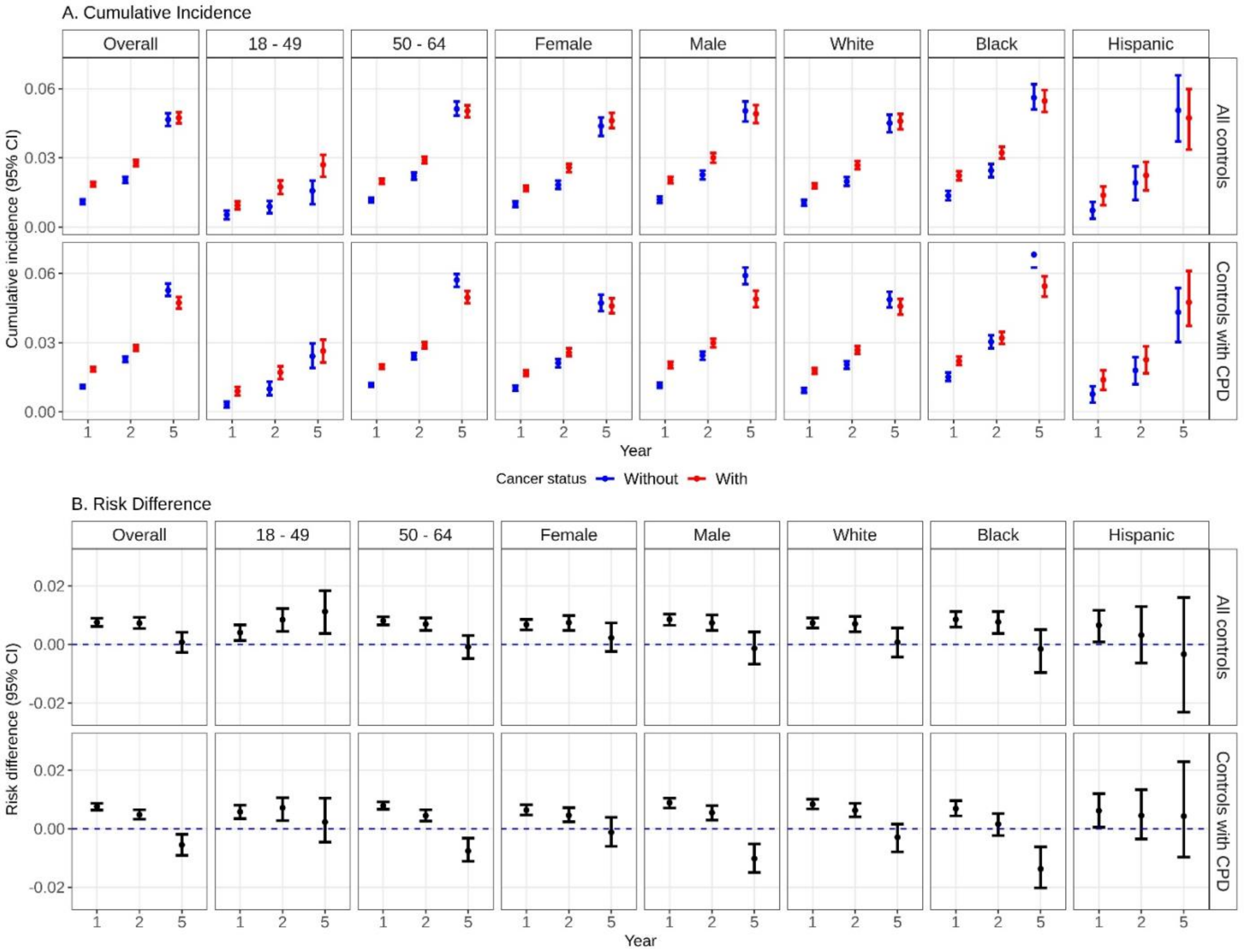

Figure S5. A. Weighted cumulative incidence (risk%) and B. weighted risk difference of dementia at years 1, 2, and 5 after baseline comparing Medicaid beneficiaries with lung cancer and their matched controls (all controls – 1st row of each panel, controls with chronic pulmonary disease – 2nd row of each panel), overall and stratified by age, sex, and race/ethnicity.

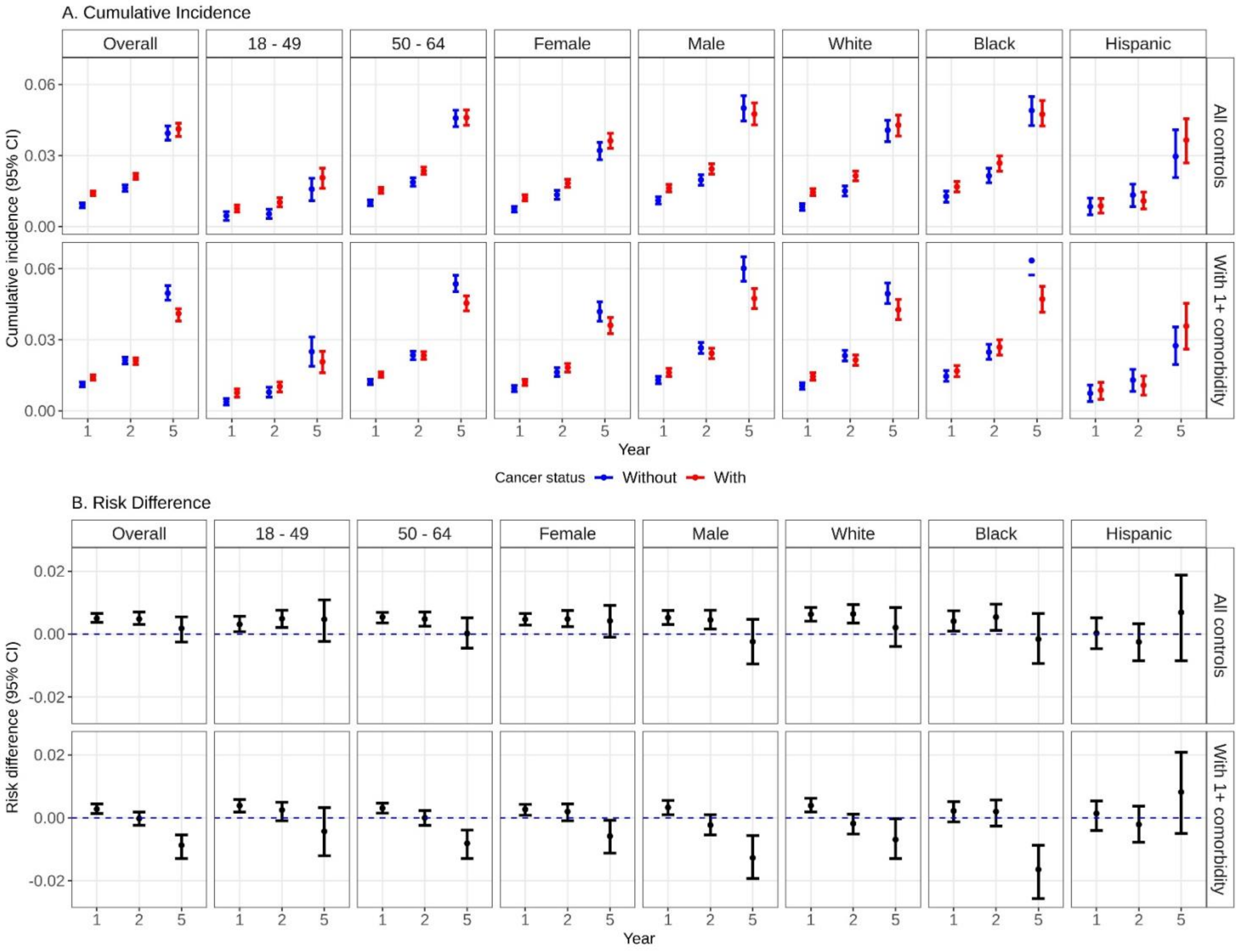

Figure S6. A. Weighted cumulative incidence (risk%) and B. weighted risk difference of dementia at years 1, 2, and 5 after baseline comparing Medicaid beneficiaries with colon cancer and their matched controls (all controls – 1st row of each panel, controls with ≥1 comorbidity – 2nd row of each panel), overall and stratified by age, sex, and race/ethnicity.

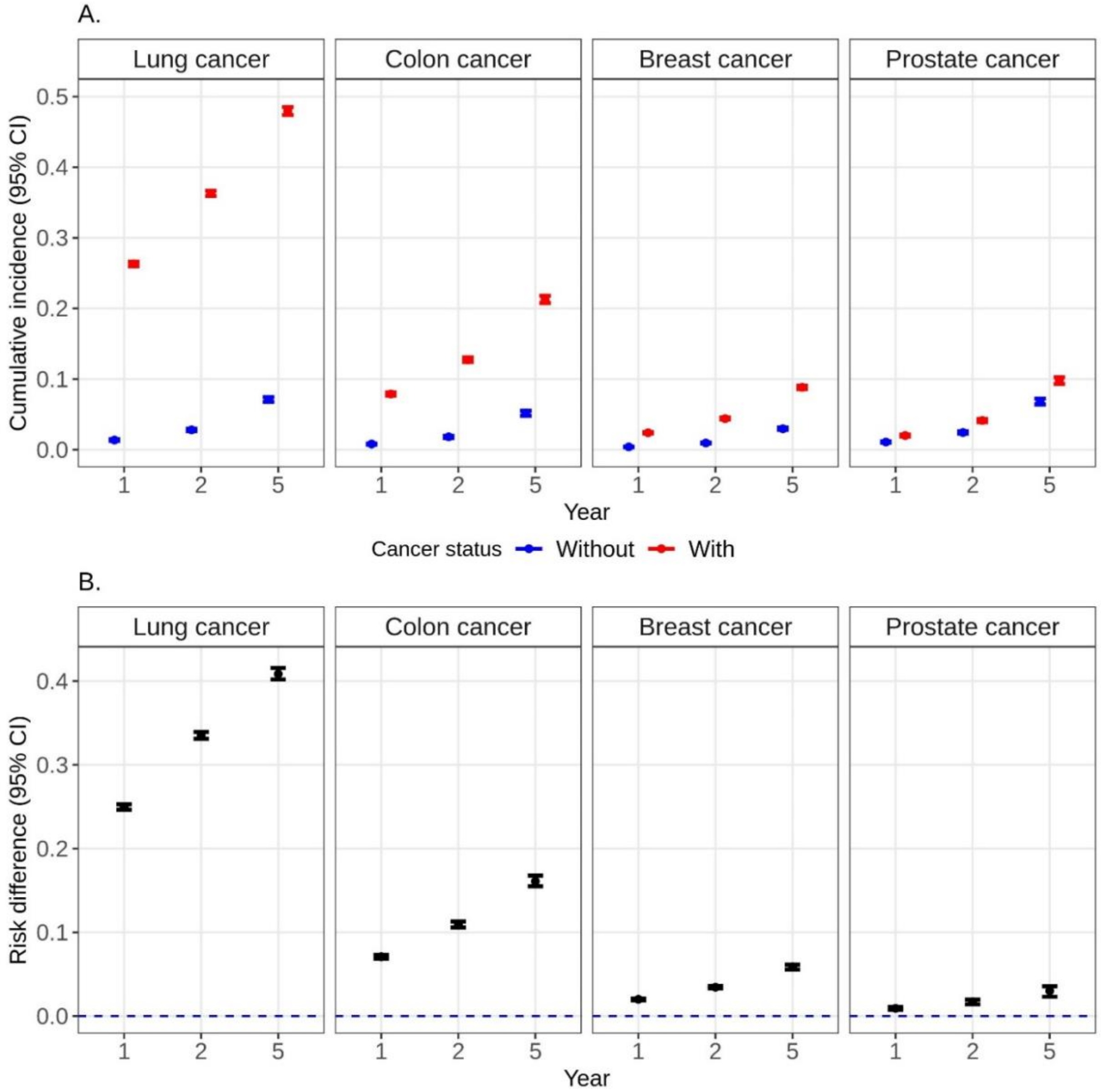

Figure S7. A. Weighted cumulative incidence (risk%) and B. weighted risk difference of death at years 1, 2, and 5 after baseline among matched Medicaid beneficiaries by cancer status.

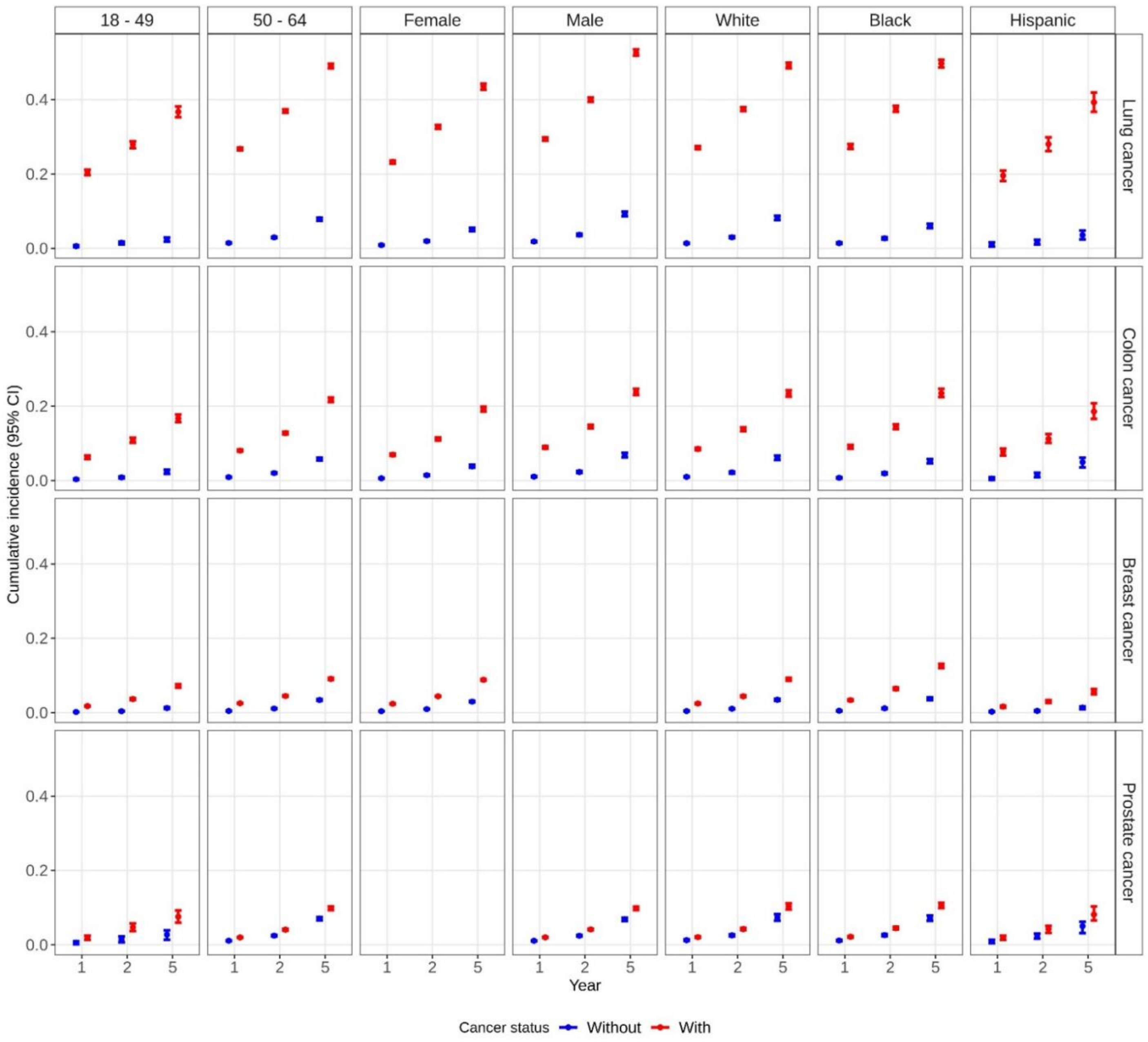

Figure S8. Weighted cumulative incidence (risk%) of death at years 1, 2, and 5 after baseline among matched Medicaid beneficiaries by cancer status, age, sex, and race/ethnicity.

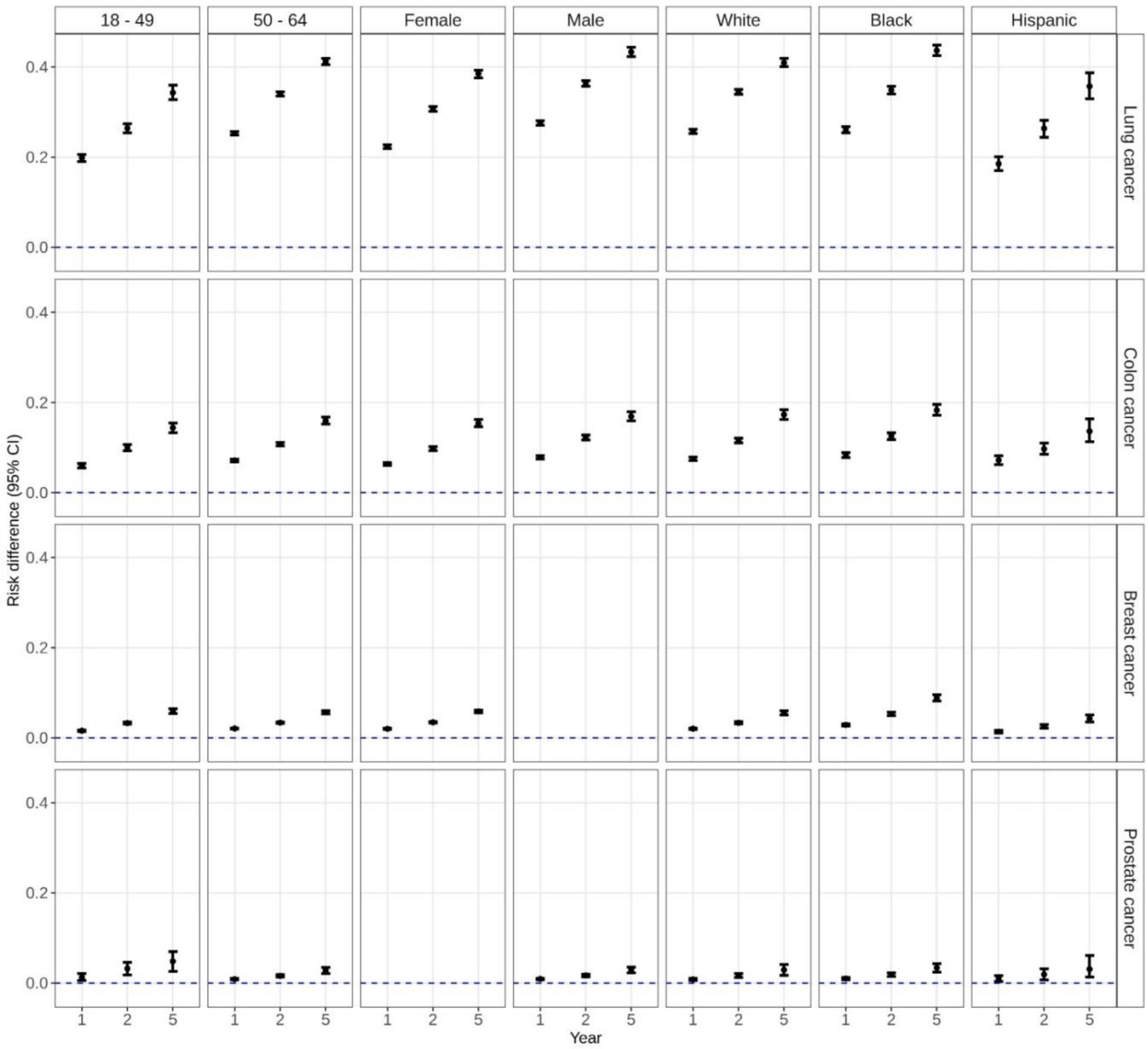

Figure S9. Weighted risk difference of death at years 1, 2, and 5 after baseline among matched Medicaid beneficiaries by cancer status, age, sex, and race/ethnicity.
